# Engaging leadership and nurse well-being: the role of the work environment and work motivation—a cross-sectional study

**DOI:** 10.1186/s12960-023-00886-6

**Published:** 2024-01-15

**Authors:** Dorothea Kohnen, Hans De Witte, Wilmar B. Schaufeli, Simon Dello, Luk Bruyneel, Walter Sermeus

**Affiliations:** 1 Research Group Work, Organizational, and Personnel Psychology, KU Leuven, Leuven, Belgium; 2 Optentia Research Unit, North-West University, Vanderbijlpark, South Africa; 3 Department of Psychology, Utrecht University, Utrecht, The Netherlands; 4 KU Leuven Institute for Healthcare Policy, KU Leuven, Leuven, Belgium

**Keywords:** Leadership, Well-being, Motivation, Work environment

## Abstract

**Background:**

Healthcare literature suggests that leadership behavior has a profound impact on nurse work-related well-being. Yet, more research is needed to better conceptualize, measure, and analyse the concepts of leadership and well-being, and to understand the psychological mechanisms underlying this association. Combining Self-Determination and Job Demands-Resources theory, this study aims to investigate the association between engaging leadership and burnout and work engagement among nurses by focusing on two explanatory mechanisms: perceived job characteristics (job demands and resources) and intrinsic motivation.

**Methods:**

A cross-sectional survey of 1117 direct care nurses (response rate = 25%) from 13 general acute care hospitals in Belgium. Validated instruments were used to measure nurses’ perceptions of engaging leadership, burnout, work engagement, intrinsic motivation and job demands and job resources. Structural equation modeling was performed to test the hypothesised model which assumed a serial mediation of job characteristics and intrinsic motivation in the relationship of engaging leadership with nurse work-related well-being.

**Results:**

Confirmatory factor analysis indicated a good fit of the measurement model. The findings offer support for the hypothesized model, indicating that engaging leadership is linked to enhanced well-being, as reflected in increased work engagement, and reduced burnout. The results further showed that this association is mediated by nurses’ perceptions of job resources and intrinsic motivation. Notably, while job demands mediated the relationship between EL and nurses’ well-being, the relationship became unsignificant when including intrinsic motivation as second mediator.

**Conclusions:**

Engaging leaders foster a favourable work environment for nursing staff which is not only beneficial for their work motivation but also for their work-related well-being. Engaging leadership and job resources are modifiable aspects of healthcare organisations. Interventions aimed at developing engaging leadership behaviours among nursing leaders and building job resources will help healthcare organisations to create favourable working conditions for their nurses.

*Trial Registration*: The study described herein is funded under the European Union’s Horizon 2020 Research and Innovation programme from 2020 to 2023 (Grant Agreement 848031). The protocol of Magnet4Europe is registered in the ISRCTN registry (ISRCTN10196901).

**Supplementary Information:**

The online version contains supplementary material available at 10.1186/s12960-023-00886-6.

## Introduction

The nursing profession is under increased pressure due to a global staff shortage and high turnover rates [1, 2]. Nurses have to cope with high workload that, combined with other work-related demands, may have severe consequences not only for their own physical and psychological health but also for their patients’ safety [3–5]. Empirical evidence suggests that leadership behavior has a profound impact on nurses’ perceived work-related strain and psychological well-being [6, 7]. For instance, poor leadership and lack of autonomy may contribute to nurse burnout, whereas recognition, rewards, and acknowledgement may enhance work-related well-being [8–10]. A recent systematic review on nursing leadership concluded that positive leadership styles (e.g., transformational leadership) and empowerment of staff foster nurses’ well-being at work [10].

Despite the widely acknowledged importance of leadership in creating healthy workplaces, the majority of leadership studies have largely neglected its impact on health-related outcomes, such as burnout and work engagement, mostly in favour of job performance or job satisfaction [7]. Even more, previous research has underestimated (inadequate) leadership as a driving factor in the development of employee well-being and ill-health, partly because of poor conceptualization, measurement, or analysis of leadership and burnout [11]. For instance, earlier studies assessed burnout rather as a one-dimensional construct by solely focusing on the emotional exhaustion dimension of the Maslach Burnout Inventory (MBI) [12]. The MBI is the most widely used instrument to assess occupational burnout; however, it has been frequently criticized because of conceptual, practical and psychometric shortcomings [13]. Similarly, most burnout research considered leadership behavior as rather narrow (e.g., by social support) instead of a comprehensive, multidimensional concept [14]. In relation to leadership, previous leadership concepts, particularly transformational leadership, have been criticized, because they lack a theoretical foundation and detailed description of the underlying processes [15, 16]. Accordingly, there is still much debate in the literature about the (motivational) underlying processes through which leaders influence employee well-being [10, 17].

In the present study, the focus is on the concept of engaging leadership (EL) [11, 18] which is measured and understood through the perceptions of nurses. Rooted in Self-Determination theory (SDT, [19]), our research builds on the premise that a resourceful workplace as perceived by employees is not only beneficial for their health but also for their work motivation. By inspiring, strengthening, connecting, and empowering employees, engaging leaders are supposed to balance their follower’s job demands and resources in such a way that they remain healthy, motivated, productive, and satisfied [11]. This resonates with research indicating that leaders, including engaging leaders, indirectly influence their followers’ well-being by shaping their perceptions of their work environment (i.e., in the form of reduced job demands and improved job resources) [6, 7, 11]. SDT further posits that employees perform and feel better when their motivation is autonomous in nature (i.e., intrinsic). A workplace where employees experience sufficient support, receive high-quality feedback, and have opportunities for professional development, therefore, provides the fuel required for optimal motivation and leads to optimal functioning and well-being [20, 21].

The overall aim of this study is to address the aforementioned conceptual and theoretical shortcomings of current leadership research by (1) using a new comprehensive measure of burnout, namely, the Burnout Assessment Tool [13, 22], (2) focusing on the concept of Engaging Leadership (EL) that is rooted in SDT, a well-established theory of human motivation, and (3) focusing on two explanatory mechanisms: perceived job characteristics (i.e., job resources and job demands) and intrinsic motivation in the relationship of leadership with nurse well-being. Drawing on the Job Demands–Resource (JD–R) leadership model and SDT, we propose an integrative model which links EL with nurses’ perceived work-related well-being (i.e., burnout and work engagement) through two explanatory mechanisms: perceived job characteristics (job demands and resources) and intrinsic motivation.

## Theoretical background and study hypotheses

### The JD–R leadership model

To explain the link between leadership behavior and nurse well-being, we use the JD–R leadership model as conceptual model, an extension of the original JD–R model [11, 23, 24]. At its core, the JD–R model proposes two psychological processes by which excessive job demands and lacking job resources are associated with well-being. First, job demands foster—via burnout—negative outcomes (i.e., health impairment process). Second, job resources yield—via work engagement—positive outcomes (i.e., motivational process). Job demands describe “aspects of the job that require sustained physical or mental effort” [23] and, therefore, are said to drain employees’ energy. By contrast, job resources are considered as the positive aspects of the job “that are either/or (1) functional in achieving work goals, (2) reduce job demands and the associated physiological and psychological costs, (3) stimulate personal growth, learning, and development’’ [23]. Job resources are assumed to have motivational potential as they may not only promote work engagement but also reduce burnout. In this study, the focus is on both the negative aspect (burnout) and positive aspect (work engagement) of job-related well-being as leadership behaviour shows to have differential relationships with these constructs [7, 25]. Burnout is defined as a work-related state of mental exhaustion, which is characterized by extreme tiredness, reduced ability to regulate cognitive and emotional processes, and mental distancing [13]. Being repeatedly confronted with heavy levels of workload and other work-related stressors, nurses are considered at a high risk of burnout [26]. In contrast, work engagement refers to a positive, fulfilling, work-related state of mind that is characterized by vigor (i.e., high level of energy and mental resilience while working), dedication (referring to a sense of significance, enthusiasm, and challenge), and absorption (being focused and happily engrossed in one’s work) [24, 27]. A large-scale prevalence study of work engagement in 30 European countries revealed that employees in human service jobs such as health care reported higher levels of work engagement than employees in other types of industries [28].

Following the JD–R leadership model, leadership is one of the unique antecedents and plays a decisive role in both the health impairment and motivational process [11, 29]. In other words, leaders who encourage and give supportive feedback, and who show recognition, may provide nurses with sufficient resources and thereby positively influence their health and well-being (i.e., the motivational process) [9, 30]. In contrast, leaders who fail to provide constructive feedback, who show less support, or who exert undue control and pressure on their staff may—due to reduced resources and increased demands—contribute substantially to feelings of stress reducing individual well-being (i.e., the health impairment process) [31]. Empirical research supports this assumption. For instance, Nielsen et al. [17] found that various work characteristics (e.g., role clarity, opportunities for development) mediated the relationship between leadership and health professionals’ well-being. In a similar vein, Schaufeli [11] reported that EL is positively associated with work engagement through job resources (e.g., task variation, role clarity) and negatively associated with burnout through reduced job demands (e.g., work overload, emotional demands). These results were further supported in a study among nursing staff [32].

### Intrinsic motivation in the JD–R leadership model

Another focus of the present study is to examine the mediating role of work motivation in the JD-R leadership model by drawing on the main premises of SDT. The focus is on one specific facet of motivation, namely, intrinsic motivation (as a form of autonomous motivation), which is defined as “the doing of an activity for its inherent satisfactions rather than for some separable consequence” [33]. If people are intrinsically motivated to perform a task, they do so for its own sake, because they perceive the task as interesting and pleasurable [33]. Following the premises of SDT, nurses will feel intrinsically motivated and healthier when they find themselves in a work environment providing them with sufficient job resources, such as autonomy, skill use, opportunities for growth and development, performance feedback. On the other hand, a demanding work environment in which nurses experience excessive job demands such as high workloads, emotional demands and a lot of bureaucracy might not only reduce their work motivation but also put them at a higher risk for burnout.

Engaging leaders are expected to behave in such a way that they fulfil their followers’ work-related basic needs [11] which, in turn, is expected to fuel intrinsic motivation [34]. Schaufeli [11] proposes four components of EL, namely, empowering, strengthening, inspiring, and connecting. These may shape nurses’ perceptions of their work environment, thereby nurturing their work motivation. First, engaging leaders empower nurses by giving them a voice and by recognizing their ownership. As a result, they will experience more autonomy and control over their own job which is likely to foster their intrinsic motivation. Second, nurses are strengthened, because engaging leaders assign them challenging tasks stimulating their talents and skills. Through strengthening, leaders foster nurses’ feeling of mastery and competence, particularly through positive feedback, which are considered as one of the prerequisites for the development of intrinsic motivation. Third, nurses are inspired to work towards an overall goal of the team or organization driven by a commitment to a vision and encouraged by their leader. The leader further acknowledges each member’s individual contribution towards the overall goal, which will increase nurses’ experience that their work is meaningful, and their contribution makes a difference. As a result, they are likely to become intrinsically motivated. Finally, engaging leaders connect their followers, for example, by fostering collaboration and a strong team spirit. In doing so, they promote a work environment characterized by a sense of security and relatedness in which nurses’ intrinsic motivation is expected to flourish. Hence, by empowering, strengthening, inspiring, and connecting, engaging leaders are considered to create favourable working conditions characterized by feelings of autonomy, competence, meaning, and relatedness which in turn will increase nurses’ intrinsic motivation. This experience is likely to result in higher levels of work engagement and well-being. Previous studies have mainly focused on the concept of transformational leadership. Research on EL is, however, relatively new and has not widely been researched yet. Nevertheless, SDT-based research generally supports this assumption [35, 36]. For instance, a meta-analytic review shows that a work environment where leaders support their employees to work autonomously is not only beneficial for the satisfaction of employees’ basic needs but also for their (intrinsic) motivation [37]. While the researchers found that leader autonomy support was positively related to intrinsic motivation, it showed, on the other hand, negative associations with employees’ distress (i.e., burnout and work stress). These findings find support by Slemp et al. [38] who conducted a meta-analytic review of 72 studies on the motivational processes and consequences of leader autonomy support in the workplace—behaviours that may be also typical of EL. Furthermore, Fernet et al. [36] showed that (transformational) leadership was significantly related to nurse well-being by contributing to favourable working conditions and intrinsic motivation.

### Objective and hypotheses

**Fig. 1:**
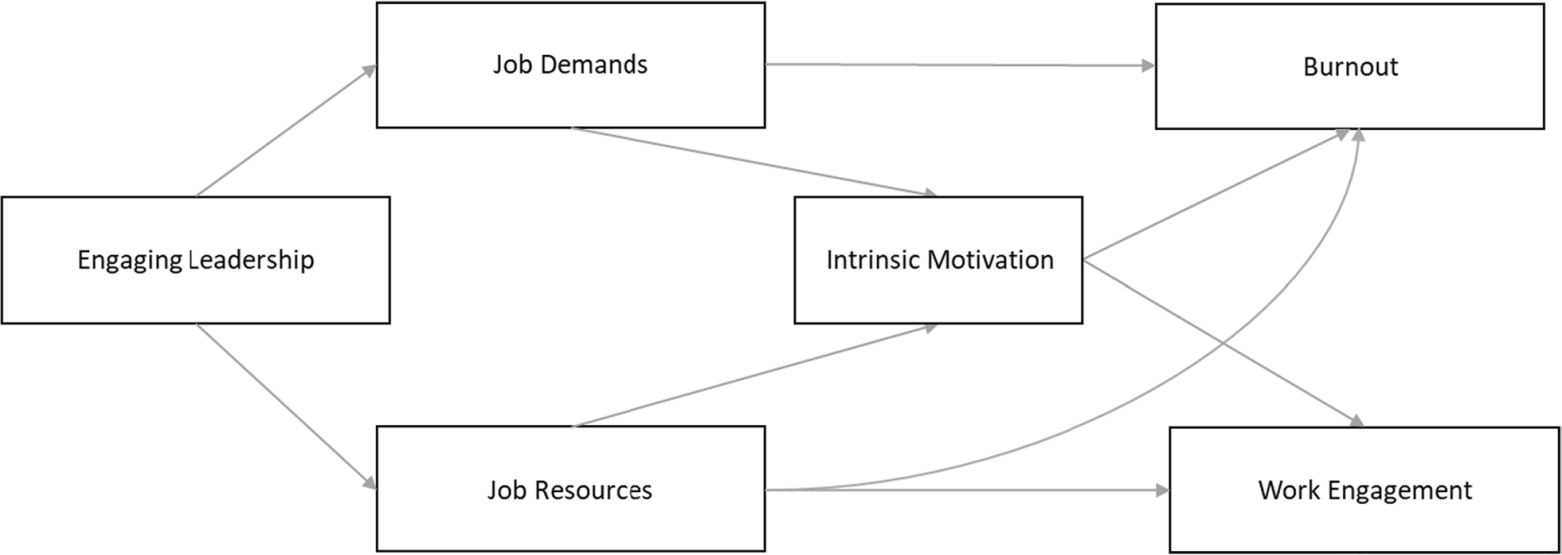
Hypothesised model

We propose that engaging leaders indirectly influence nurse well-being and intrinsic motivation by providing more job resources and by reducing job demands. Focusing on the mediating role of job characteristics and intrinsic motivation, considering that engaging leaders (a) support nurses to balance their job resources and job demands and (b) nurture their intrinsic motivation through improved working conditions, it is expected that engaging leaders influence nurses’ work-related perceived strain and well-being (i.e., reduced levels of burnout and increased work engagement). The proposed model is shown in Fig. 1. The hypotheses are as follows:

#### H1

Job demands mediate the relationship of EL with 1a) burnout and 1b) work engagement.

#### H2

Job resources mediate the relationship of EL with 2a) work engagement and 2b) burnout.

#### H3

The relationship of EL with intrinsic motivation is mediated by 3a) job demands and 3b) job resources.

#### H4

Job demands and intrinsic motivation mediate the relationship of EL with 4a) burnout and 4b) work engagement.

#### H5

Job resources and intrinsic motivation mediate the relationship of EL with 5a) work engagement and 5b) burnout.

## Methods

### Design and setting

The present study employed a cross-sectional data set of 1117 direct care nurses from 13 general acute care hospitals in the Flemish (i.e., Dutch speaking) region of Belgium.

### Data collection

The data used for this study were collected between May 2022 and August 2022 in the context of the Horizon 2020 funded Magnet4Europe project [39]. The project aims to investigates doctors’ and nurses’ perceptions towards leadership, their working environment, motivation, and well-being. In the present study, we report only on the data collected from the nurses that participated in the survey. Data were processed in line with the General Data Protection Regulation 2016/679 of the European Union (EU, [40]). More information on the data collection process in general can be found in Kohnen et al. [21].

### Participants

A total of 5889 registered nurses were invited to participate in the survey. Registered nurses were eligible to participate if they had direct patient contact and worked on adult inpatient units, including intensive care units (ICU) and the emergency room (ER). Of the 5445 questionnaires sent, 1374 were filled in and returned, which yielded a response rate of 25%. To keep the work situation rather constant and comparable, the target population for this study included nursing professionals in the same job level, i.e., direct care nurses [41]. Accordingly, the final data set consisted of 1117 observations. Regarding the demographic characteristics, 82% of the nursing staff were female, the average age was 40 years (sd = 12) and they had been working in their current hospital for 15 years on average (sd = 11). Direct care nurses from all types of departments were included in the study. The majority was working in intensive care (25.3%), followed by nurses active on surgical (18.5%), internal (17.5%), and geriatric (12.5%) units.

### Measures

A description of all measures, based on existing scales available in Dutch, and their internal consistencies (Cronbach’s alpha) are described below. Unless stated otherwise, all variables were scored on a five-point Likert scale ranging from 1 (never) to 5 (always).

#### Engaging leadership

Employees perception of engaging leadership was measured with the 12-item Engaging Leadership scale [11, 29]. The instrument assesses the four core dimensions of EL with three items each. In the Dutch version of the survey instrument, the term 'leidinggevende' was used to refer to 'supervisor.' Example items were: “My supervisor delegates tasks and responsibilities to team members” (strengthening), “My supervisor encourages among team members to cooperate” (connecting), “My supervisor encourages team members to voice their opinions” (empowering), and “My supervisor is inspiring” (inspiring). The value of Cronbach’s alpha for the total scale of EL was 0.96.

#### Job demands and resources

The questionnaire captured a set of five job demands and seven job resources which were mostly derived from the Energy Compass psychometric instrument [42]. Job resources included autonomy (4-items), role clarity (2-items), performance feedback (3-items), skill use (1-item), opportunities for growth and development (1-item), task variety (1-item), and value congruence (1-item). Job demands included role conflicts (3-items), workload (4-items), red tape (1-item), emotional dissonance (1-item), and emotional demands (1-item). For every respondent, composite scores of all job demands and job resource measures were generated, i.e., scores on the seven job resources as well as on the five job demands were each combined into one mean value. The value of Cronbach’s alpha for both scales were 0.84.

#### Intrinsic motivation

Intrinsic motivation was assessed with three items from the Work Extrinsic and Intrinsic Motivation Scale (WEIMS, [43]), rated on a five-point Likert scale ranging from completely disagree (1) to completely agree (5). The header for the scale was: “Please indicate to what extent each of the following items corresponds to the reasons why you are presently involved in your work.” An example item was “Because I derive much pleasure from learning new things.”. The value of Cronbach’s alpha for this scale was 0.83.

#### Burnout

Burnout was measured using the short version of the Burnout Assessment Tool (BAT), a novel self-report questionnaire [13]. The short version of the BAT [44] consists of 12-items that assess the presence of the four core burnout syndromes, with three items each: “At work, I feel mentally exhausted” (exhaustion), “I feel a strong aversion towards my job” (mental distance), “At work, I may overreact unintentionally” (emotional impairment), and “When I’m working, I have trouble concentrating” (cognitive impairment). The value of Cronbach’s alpha for the total burnout scale was 0.90.

#### Work engagement 

Work engagement was measured using three items from the Dutch version of the Utrecht Work Engagement Scale (UWES, [45]). The UWES-3 assesses the three core dimensions of work engagement, with one item each. An example item was: “At my work, I feel bursting with energy” (vigor). The value of Cronbach’s alpha for this scale was 0.84.

#### Covariates

To rule out the possibility that the associations can be explained by relevant third variables, we controlled for the impact of the hospital, gender, and age.

### Data analysis

Data analysis was done using SPSS Version 28 [46] and Mplus Version 8.6 [47]. To estimate our model, we performed structural equation modeling (SEM) with maximum likelihood estimation methods. In line with the two-stage approach proposed by Anderson and Gerbing [48], we first tested the measurement model and in a second step the hypothesised structural model. The measurement model was tested using Confirmatory Factor Analysis (CFA). We considered a number of fit indices to assess how well the hypothesised measurement model fits to the data [49, 50]: Comparative Fit Index (CFI), Tucker–Lewis Index (TLI), the Root Mean Square Error of Approximation (RMSEA) and the Standardised Root Mean Residual (SRMR). For the CFI and TLI, values above 0.90 indicated an adequate, values above 0.95 an even better model fit. For the RMSEA values should ideally be below 0.06 and for SRMR below 0.08, respectively.

Next, the hypothesised structural model was evaluated. Bootstrapping was applied with a resample procedure of 1000 bootstrap samples to determine the point estimate and bias-corrected and accelerated 95% confidence interval (CI) of the total and specific indirect effect [51, 52]. Bootstrapping is recommended as the indirect effect (the product of the coefficients of the predictor and mediator variable) is not normally distributed [53]. A bootstrapped confidence interval (lower level of confidence interval − upper level of confidence interval, LLCI − ULCI) that does not include the null value is indicated as statistically significant. As shown in Fig. 1, we did not hypothesise direct relationships between EL and intrinsic motivation, or between EL and burnout and work engagement. Rather, it was assumed that these relationships are explained through job demands and resources. Following Hayes [51], a significant association between the predictor and the outcome is neither a necessary nor a sufficient condition of mediation. Yet, the direct paths from EL to each outcome variable were also added in the SEM. Any direct associations among the variables included in this study are documented in the supplementary material of this study (Additional file 1: Appendix 1).

## Results

Table 1 provides information on the means, standard deviations, and correlations for all constructs. Correlations among the variables included were statistically significant and in the expected direction. In addition, we used correlational analyses to verify the associations between the sociodemographic variables (i.e., age and gender) and the variables of our model. Age significantly correlated weakly with intrinsic motivation (− 0.139, *p* < 0.001), work engagement (0.086, *p* < 0.001), and burnout (− 0.104, *p* < 0.001), while gender showed no significant correlations with any of the variables. In addition, when including these variables as controls, the hypothesised associations did not change substantially. The same was observed when including hospitals as covariate. Therefore, to aid clarity, we report the most parsimonious analysis without including hospitals, age and gender as control variables [54].

**Table 1:** Means, standard deviations, and Pearson correlations among study variables

Concept (# of items)	Mean	SD	Correlations							
			1	2	3	4	5	6	7	8
1. Gender	–	–	1							
2. Age	40	12	− 0.059*	1						
3. Engaging Leadership (12)	3.49	0.81	0.018	0.011	1					
4. Job Demands (10)	3.30	0.48	− 0.039	− 0.009	− 0.302**	1				
5. Job Resources (13)	3.40	0.48	0.039	0.032	0.565**	− 0.405**	1			
6. Intrinsic Motivation (3)	3.80	0.62	0.047	− 0.139**	0.247**	− 0.213**	0.456**	1		
7. Work Engagement (3)	3.49	0.67	0.061	0.086**	0.341**	− 0.348**	0.527**	0.497**	1	
8. Burnout (12)	2.18	0.57	0.023	− 0.104**	− 0.315**	0.589**	− 0.468**	− 0.347**	− 0.591**	1

**Correlation is significant at the 0.01 level (two-tailed), gender was coded 1 = male and 2 = female. The range of scale for all variables is 1–5

### Confirmatory factor analysis

A CFA was conducted to evaluate the measurement model which consisted of six correlated latent variables: engaging leadership (a second-order factor represented by its four dimensions strengthening, connecting, empowering, and inspiring; each represented by their three corresponding items), job demands (a first-order factor represented by items assessing workload, role conflicts, emotional demands, bureaucracy, and emotional dissonance), job resources (a first-order factor represented by items assessing autonomy, performance feedback, role clarity, task variety, skill use, opportunities for growth and development, and value congruence), intrinsic motivation (a first-order factor represented by its three items), burnout (a second-order factor represented by its four dimensions exhaustion, mental distance, cognitive impairment, and emotional impairment; each represented by their three corresponding items), and, finally, work engagement (a first-order factor represented by its three items). The results of the CFA indicated a good fit of our hypothesised measurement model, with χ^2^ (797) = 2566.703, CFI = 0.93, TLI = 0.93, RMSEA = 0.05, and SRMR = 0.06. All indicators showed significant factor loadings on their respective latent factors (*p* < 0.001) with λ values ranging from 0.38 to 0.93. A reliable measurement model was, therefore, obtained.

### Results of the mediation analysis

**Fig. 2:**
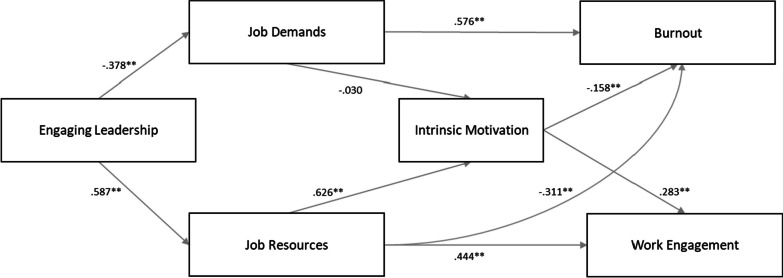
Structural model of intrinsic motivation in the JD-R leadership model. Coefficients represent standardized estimates. **p < 0.001

First, we tested the main premises of the JD-R model (cfr. Fig. 2). Our results indicted a positive relationship of job demands with burnout (*β* = 0.576, *p* < 0.001) as well as a positive relationship of job resources with work engagement (*β* = 0.444, *p* < 0.001). In addition, job resources were negatively related to burnout (*β* = − 0.311, *p* < 0.001).

Table 2 provides information on the indirect relationships and mediating effects. Our first set of hypotheses aimed at testing the main assumptions of the JD-R leadership model, i.e., job demands and job resources are expected to mediate the relationship of EL with nurse well-being (burnout and work engagement). In relation to H1a, we observed an indirect effect of EL on burnout via job demands (*β* = − 0.218) which was statistically significant according to the bootstrap CI 95% (− 0.272, − 0.176). With regard to H1b, our results showed an indirect effect of EL on work engagement via job demands (*β* = 0.064) which was statistically significant according to the bootstrap CI 95% (0.035, 0.100). These results confirm H1a and H1b.

**Table 2:** Indirect effect of engaging leadership on burnout and work engagement through job demands, job resources, and intrinsic motivation

Hypothesis	Predictor	Mediator	Outcome	β	ρ	LLCI	ULCI
1a	Engaging Leadership	Job demands	Burnout	− 0.218	0.000	− 0.272	− 0.176
1b	Engaging Leadership	Job demands	Work engagement	0.064	0.000	0.035	0.100
2a	Engaging Leadership	Job resources	Work engagement	0.260	0.000	0.192	0.342
2b	Engaging Leadership	Job resources	Burnout	− 0.183	0.000	− 0.262	− 0.121
3a	Engaging Leadership	Job demands	Intrinsic motivation	0.011	0.457	− 0.018	0.040
3b	Engaging Leadership	Job resources	Intrinsic motivation	0.368	0.000	0.286	0.457
4a	Engaging Leadership	Job demands and intrinsic motivation	Burnout	− 0.002	0.461	− 0.007	0.003
4b	Engaging Leadership	Job demands and Intrinsic motivation	Work engagement	0.003	0.462	− 0.005	0.012
5a	Engaging Leadership	Job resources and intrinsic motivation	Work engagement	0.104	0.000	0.070	0.144
5b	Engaging Leadership	Job resources and intrinsic motivation	Burnout	− 0.058	0.000	− 0.095	− 0.032

β = Standardized beta, LLCI = lower level of bootstrap confidence interval, ULCI = upper level of bootstrap confidence interval

With regard to H2a, our results showed an indirect effect of EL on work engagement via job resources (*β* = 0.260), which was statistically significant according to the bootstrap CI 95% (0.192, 0.342). In relation to H2b, the results further indicated an indirect effect of EL on burnout via job resources (*β* = − 0.183). This effect was statistically significant according to the bootstrap CI 95% (− 0.262, − 0.121). Accordingly, these results support H2 and, therefore—taken together with H1a—the main assumptions of the JD-R leadership model.

Next, it was hypothesised that the relationship of EL with intrinsic motivation is mediated by job demands (H3a) and job resources (H3b). The observed indirect association between EL and intrinsic motivation via job demands (*β* = 0.011) was not statistically significant according to the bootstrap CI 95% (− 0.018, 0.040). Regarding H3b, our results showed an indirect effect of EL on intrinsic motivation via job resources (*β* = 0.368) which was statistically significant according to the bootstrap CI 95% (0.286, 0.457). In short, while the results do not support H3a, they do confirm H3b.

Our next set of hypotheses stated that job demands and intrinsic motivation mediate the relationship of EL with burnout (H4a) and work engagement (H4b). We found that the indirect effect of EL on burnout via job demands and intrinsic motivation (*β* = − 0.002) was not statistically significant according to the bootstrap CI 95% (− 0.007, 0.003). In relation to work engagement, we observed an indirect effect of EL on work engagement via job demands and intrinsic motivation (*β* = 0.003). This effect was not statistically significant according to the bootstrap CI 95% (− 0.005, 0.012). These results do not confirm H4.

Finally, it was hypothesised that job resources and intrinsic motivation mediate the relationship of EL with work engagement (H5a) and burnout (H5b). The results show that EL had an indirect on work engagement via job resources and intrinsic motivation (*β* = 0.104). This effect was statistically significant according to the bootstrap CI 95% (0.070, 0.144). In addition, we found that EL had an indirect effect on burnout via job resources and intrinsic motivation (*β* = − 0.058), which was statistically significant according to the bootstrap CI 95% (− 0.095, − 0.032). Consequently, these results support H5.

## Discussion

Based on the JD-R leadership model and SDT, we tested an integrative model which links engaging leadership with nurses’ perceived work-related well-being (i.e., burnout and work engagement) through two explanatory mechanisms: perceived job characteristics (job demands and resources) and intrinsic motivation. The results from the structural equation modeling largely support this model: engaging leaders can shape nurses’ perceptions of their work and create a work environment that is characterized by more resources and fewer demands. Particularly, by providing nurses with sufficient job resources such leaders do not only nurture their intrinsic motivation but also foster their perceived well-being (i.e., reduced levels of burnout and increased work engagement).

Overall, our results are in line with what has been suggested by previous studies: job resources appear to be a crucial factor for nurse work engagement, whereas job demands remain an essential driver of burnout. Consistent with the JD-R leadership model, we found that job demands mediated the relationship between EL and burnout (H1a). Similarly, job demands mediated the relationship between EL and work engagement (H1b) indicating that engaging leaders can rule out the detrimental effect of job demands on nurse well-being. Job resources, on the other hand, mediated the relationship of EL with work engagement (H2a) but also with burnout (H2b). These results illustrate that engaging leaders indirectly influence nurses’ well-being by shaping their perceived job resources and job demands. By empowering, inspiring, strengthening, and connecting, engaging leaders create a more favourable work environment for nurses characterized by reduced demands (less workload, emotional demands, role conflicts) and increased resources (sufficient autonomy, task variety, skill use and feedback). As a result, nurses will not only experience higher levels of work engagement, also, they are less likely to experience feelings of burnout. These results are in line with the findings reported in previous studies [11, 32]. For instance, by including a wide variety of job demands (i.e., emotional demands, role conflict, work overload) and job resources (i.e., job control, task variety, skill use), Schaufeli [11] found that followers’ perceptions of their work characteristics mediated the relationship between EL and employee well-being.

In addition, our findings underscore the importance of work motivation in the JD-R leadership model. Through increased job resources, engaging leaders seem to foster nurses’ intrinsic motivation (H3b). Particularly, the results indicate that nurses who feel empowered, inspired, strengthened, and connected through EL behavior are more likely to perceive sufficient autonomy and a strong connection with their team. In addition, they will believe in their ability to master their work and to contribute meaningfully to the workplace. Consequently, they will find themselves in a work environment in which their intrinsic motivation is expected to flourish. Following SDT, these results suggest that engaging leaders foster—via job resources—an internalization process in nurses, inducing a sense of enjoyment and satisfaction while performing their job [55]. Even more, as illustrated in the current sample, nurses are likely to feel more engaged at work (H5a), while they are less likely to develop feelings of burnout (H5b). This study adds to the accumulating evidence of the JD-R model and SDT by showing that leadership behavior has a profound impact on nurses’ perceptions of their work environment, their work motivation, and work-related well-being.

Contrary to our expectations, we found no mediating effect of job demands and intrinsic motivation in the relationship of EL with nurses’ burnout (H4a) and work engagement (H4b). Although job demands mediated the relationship between EL and nurses’ well-being, the relationship became unsignificant when including intrinsic motivation as second mediator. Overall, these findings align with a recent study by Kohnen et al. [21]. In a sample of 1729 direct care nurses in Belgium, the authors investigated the mediating role of intrinsic motivation in the relationship of job demands and job resources with nurse work-related well-being. The results showed that intrinsic motivation did not mediate the relationship of job demands with nurse well-being. In a similar vein, intrinsic motivation showed no associations with job demands in general. In relation to the current study, our findings prompt further exploration into the specific aspects of EL, as it may not be effectively predicting the associations of job demands and intrinsic motivation with nurses' work-related well-being. Yet, another possible explanation is that the relationship between job demands and nurse well-being is influenced by other types of motivation, such as external regulation, as a form of controlled motivation. SDT arranges different forms of motivation along a continuum of self-determination, ranging from more controlled to more autonomous motivation. While the most autonomous form of motivation, i.e., intrinsic motivation, is at one extreme of the continuum, the most controlled form of motivation (i.e., external regulation) is at the other end of the spectrum. External regulation occurs when employees engage in a certain behavior or activity for purely instrumental reasons, such as to obtain rewards, to avoid punishment, or simply because they are being pressured by demands. Indeed, previous research and meta-analytic evidence demonstrated that external regulation, and particularly amotivation (i.e., lack of motivation, employees shows no interest or engagement in performing a task), exerted only negative influence on employee functioning, leading to distress and burnout [36, 37]. This may suggest that autonomous and controlled motivation or amotivation are inversely associated with employees' psychological functioning. However, empirical evidence on the individual effects of the different motivational types is scarce and more research is needed to fully understand employees’ motivation in the workplace [37].

### Limitations

Some limitations of the present study need to be mentioned. First, this study employed a cross-sectional design which precludes to establish definite conclusions about causal relationships between the variables. Future studies could revalidate the model using longitudinal designs. Second, we observed a relatively low participation rate (25%) in our sample which raises concerns around sampling bias. While the COVID-19 pandemic and the associated survey fatigue among health professionals might be an explanation for the low response rates [56], research on nursing staff seems to report similar if not even lower response rates of 10–15% [57]. Similarly, our sample consisted of direct care nurses working in Belgian hospitals (*n* = 13), which puts some limitation on the generalization of our findings. While the homogeneous sample minimizes the influence of contextual factors, allowing for a straightforward test of our hypotheses, the results should be interpreted with caution. A replication of our study among other health professionals, such as physicians, or in other countries that are part of the Magnet4Europe study [39], as well as over time would strengthen the external validity of our findings. Third, several job demands and resources were measured using few or even single items scales from validated instruments. Internal consistencies of the scales, however, were beyond the usual criterion of 0.70, with a value of α = 0.84 for both scales. While scholars in occupational research seem to prefer multiple-item over single-measures, a recent study confirmed the validity of several single-item measures, such as job control [58]. Another limitation in relation to our measures is that employees’ perceptions of EL have been measured instead of surveying supervisors themselves. Future research might benefit from assessing leadership behavior by surveying non-followers. Similarly, all concepts included in this study were obtained through self-reports. As such, the strength of the effects reported herein may have been biased due to common-method variance or because of the wish to answer consistently [59]. This may be resolved in future research by including “objective” indicators of work characteristics, such as nurse staffing levels, nurse–patient ratios or average hours worked per week. Finally, while the quantitative nature of this study offers limited insights into the local context, future investigations could enhance understanding by incorporating qualitative research methods, such as interviews and focus groups. Notably, in the context of the Magnet4Europe study, interviews and focus groups have been performed among nursing staff which might offer valuable insights into their perceptions of the clinical work environment.

### Implications for practice

Despite the aforementioned limitations, our study has a few practical implications for healthcare organizations. First, the results of this study confirm that EL indirectly influences nurses’ well-being by shaping their perceptions of their working environment. In particular, when leaders provide nurses with sufficient job resources, such as autonomy, feedback, and opportunities for development, nurses are likely to become intrinsically, i.e., autonomously motivated at work which seem to be beneficial in two ways: first, it will foster their work engagement, and second, it will prevent them from burning out. The findings illustrate that engaging leaders are key agents in shaping the clinical work environment and in creating a resourceful atmosphere that is needed to improve nurses’ motivation and well-being. Accordingly, healthcare organizations may benefit from investing into training programs that support nurse leaders in developing EL behavior. As a matter of fact, research on leadership training programs shows that leadership behavior promoting favourable working conditions, autonomous motivation, and well-being can be learned [60–62]. For instance, a recent intervention study proposed a 6-day training program on EL designed in co-creation between senior leaderships and team leaders [61]. Peer consultation and personal coaching were offered between training days to support the integration of the program. The findings showed that training managers in EL behaviour was associated with improved business results, lower absenteeism, and, most importantly, improved well-being [61]. Our findings further indicate that job demands (such as heavy workloads, emotional demands, and role conflict) are an important antecedent of burnout, whereas job resources (such as autonomy, performance feedback, role clarity) are key predictors of nurse intrinsic motivation and work engagement. Healthcare organizations are, therefore, advised to invest in initiatives aimed at reducing job demands and increasing job resources. Consistent with the JD-R model, Bakker et al. [63] suggest a combination of trainings and workshops, where participants learn about possible ways to actively modify job demands and resources in their job (i.e., job crafting). Research has shown that such job crafting interventions can be very effective for employees in optimizing their perceptions of their work environment [64]. To summarize, by promoting EL, healthcare organizations may create resourceful environment for nursing staff which is not only beneficial for their work motivation but also for their overall well-being.

## Conclusion

Drawing on the JD-R leadership model and SDT, the present study tested an integrative model which links EL with nurse work-related well-being (i.e., burnout and work engagement) through two explanatory mechanisms: perceived job characteristics (job demands and resources) and intrinsic motivation. The findings provide support for the hypothesised model, suggesting that EL is associated with increased well-being (i.e., work engagement and burnout) by contributing to favourable perceptions of job characteristics (more resources and less demands) and intrinsic motivation in nurses.

### Supplementary Information

**Additional file 1:** **Appendix 1: Table S1.** Direct effects.

## Data Availability

Individual participant data that underlie the results reported in this article, after deidentification, will be shared with researchers who provide a methodologically sound proposal.

## Abbreviations

CFA: Confirmatory factor analysis
EL: Engaging leadership
JD–R model: Job demands-resources model
SDT: Self-determination theory
SEM: Structural equation modeling
