# Supplementary material for: Engaging leadership and nurse well-being: the role of the work environment and work motivation—a cross-sectional study
Source: Hum Resour Health. 2024 Jan 15;22:8. doi: 10.1186/s12960-023-00886-6 (PMC10788988; doi:10.1186/s12960-023-00886-6)
Supplement: Supplementary file 1 — Additional file 1: Appendix 1: Table S1. Direct effects. [file 12960_2023_886_MOESM1_ESM.docx]

Appendix 1

Table S1: Direct effects

| **Predictor** | **Outcome** | **β** | **ρ** | **LLCI** | **ULCI** |
| --- | --- | --- | --- | --- | --- |
| Job Resources | Burnout | -0.311 | 0.000 | -0.424 | -0.201 |
| Job Demands | Burnout | 0.576 | 0.000 | 0.499 | 0.654 |
| Intrinsic Motivation | Burnout | -0.158 | 0.000 | -0.236 | -0.082 |
| Engaging Leadership | Burnout | 0.051 | 0.225 | -0.028 | 0.134 |
| Job Resources | Intrinsic Motivation | 0.626 | 0.000 | 0.532 | 0.734 |
| Job Demands | Intrinsic Motivation | -0.030 | 0.454 | -0.106 | 0.051 |
| Engaging Leadership | Intrinsic Motivation | -0.104 | 0.048 | -0.213 | -0.003 |
| Engaging Leadership | Job Demands | -0.378 | 0.000 | -0.439 | -0.314 |
| Engaging Leadership | Job Resources | 0.587 | 0.000 | 0.522 | 0.652 |
| Job Resources | Work Engagement | 0.444 | 0.000 | 0.330 | 0.557 |
| Job Demands | Work Engagement | -0.170 | 0.000 | -0.259 | -0.098 |
| Intrinsic Motivation | Work Engagement | 0.283 | 0.000 | 0.190 | 0.368 |
| Engaging Leadership | Work Engagement | -0.004 | 0.930 | -0.095 | 0.086 |
